# Special Issue: Immune Dysfunction in Uremia

**DOI:** 10.3390/toxins13010070

**Published:** 2021-01-19

**Authors:** Gerald Cohen, Raymond Vanholder

**Affiliations:** 1 Department of Nephrology and Dialysis, Medical University of Vienna, A-1090 Vienna, Austria; 2 Nephrology Section, Department of Internal Medicine and Pediatrics, University Hospital Ghent, Corneel Heymanslaan, 10, B9000 Gent, Belgium; Raymond.Vanholder@ugent.be

This Special Issue of *Toxins* focusses on the interconnected factors interfering with the immune response in uremic patients. Inflammation-induced cardiovascular disease and infections are the two leading causes for the high risk of morbidity and mortality among patients with chronic kidney disease (CKD), and contribute to the diminished functions and altered apoptosis of cells of the innate and adaptive immune response. Impaired glomerular filtration and tubular excretion result in the retention of uremic toxins. They can pre-activate the immune system but cause at the same time a lack of responsivity when facing infection or other external stimuli. Uremic retention solutes are classified according to their physicochemical characteristics into small water-soluble molecules, middle molecules, and protein-bound molecules [1]. The intestinal microbiome is an additional source of uremic toxins. Furthermore, proteins post-translationally modified in the uremic milieu may adversely affect the immune response. In addition to a reduced glomerular filtration, the disturbed metabolic activities of the kidney contribute to a compromised immune system.

The review “Immune Dysfunction in Uremia 2020” [2] in this Special Issue describes cardiovascular disease and infections as the main causes of death in uremia, and summarizes the impact of impaired immune cells such as neutrophils with elevated formation of neutrophil extracellular traps and the involvement of toll-like receptors, inflammasomes, autophagy and the complement system on immune dysfunction. Furthermore, the impact of deranged metabolic kidney functions, such as the production of erythropoietin, calcitriol, and renin, on uremic toxicity are discussed.

Based on their CD14/CD16 expression profile, three morphologically and functionally different populations of monocytes can be distinguished. “Intermediate monocytes” (CD14++/CD16+) have pronounced pro-inflammatory properties and their proportion is an important predictor of mortality risk in HD patients [3]. A change in the relative numbers of the three different populations, but also altered features of the individual cells, can contribute to the dysregulated immune response in uremia. In a review in this Special Issue, Girndt et al. [4] emphasize the differences in uremic monocytes in terms of surface molecule expression, production of cytokines and mediators, and function, as compared to the monocytes of healthy persons.

Like innate immunity, acquired immune deficiency contributes to the high morbidity and mortality of end-stage kidney disease (ESKD) patients [5]. In this Special Issue, Betjes et al. [6] summarize the current data about uremia-associated ageing of the thymus and its role in dysfunctional adaptive immune responses. The main feature of immunological ageing is naïve T cell lymphopenia in conjunction with the expansion of highly differentiated memory T cells, with a pro-inflammatory phenotype destabilizing atherosclerotic plaques and adding to the inflammatory state.

Oxidative stress increases in parallel with the development of CKD [7]. Markers of oxidative stress predict the survival of HD patients [8]. Moreover, antioxidant systems are compromised in CKD patients and deteriorate gradually with the degree of kidney failure [9]. Nuclear factor erythroid 2-related factor 2 (Nrf2) regulates the expression of antioxidant proteins that protect against oxidative damage. Considering that genetic features play an important role in the development and prognosis of CKD, Jerotic et al. [10] investigated the association between the polymorphism in Nrf2, superoxide dismutase, and glutathione peroxidase genes, and showed that polymorphisms in these genes are associated with the development of ESKD, and can predict survival.

Toll-like receptors are pattern recognition receptors that, together with inflammasomes, detect and react to highly conserved motifs on pathogens (pathogen-associated molecular patterns), and to substances released upon cell injury or stress (damage-associated molecular patterns; DAMPs). Uremic endothelial cells may be involved in the activation of innate immunity, but they may also be damaged by this immune activation. Uremic endothelial cells produce DAMPs, which bind to specific pattern recognition receptors expressed in numerous cells, including endothelial cells, and induce the expression of adhesion molecules, the production of proinflammatory cytokines and an increased production of reactive oxygen species in endothelial cells, establishing a link between immunity and inflammation. In this Special Issue, Diaz-Ricart et al. [11] review the connection between endothelial damage, inflammation and defective immunity in uremia.

Inflammasomes are multiprotein complexes involved in innate immunity, and regulate caspase-dependent inflammation [12]. They are assembled by pattern recognition receptors after the detection of pathogens or danger signals in host cells. Dysregulated inflammasome activity is associated with hereditary and acquired inflammatory disorders [13]. Hypervolemia (fluid overload) correlates with cardiovascular risk factors in patients with CKD [14], and inflammation is usually higher in hypervolemic hemodialysis (HD) patients compared to normovolemic patients. Hypervolemia and inflammation are both independent risk factors for mortality, with a cumulative risk profile [15]. In this issue, Ulrich et al. [16] measured the hypervolemic activation of peripheral blood mononuclear cells of HD patients, with specific emphasis on the NLRP3 inflammasome response. They found that hypervolemia does not activate the NLRP3 inflammasome, implying that endotoxemia is not a major contributor to inflammation in hypervolemic HD patients.

The human immune system is critical in maintaining homeostasis with the resident microbiota. On the other hand, resident microbes influence the human immune response [17]. Under inflammatory conditions in CKD, uremic toxins of bacterial sources disrupt the intestinal barrier. Those uremic toxins activate immune cells in the circulation. In this Special Issue, Glorieux et al. [18] describe the pathophysiological effects of intestinally generated end-products of the bacterial metabolism, such as *p*-cresol, trimethylamine, and hydrogen sulfide. Furthermore, Espi et al. [19] review the latest emerging results linking the pathophysiology of CKD-associated immune dysfunctions with the accumulation of microbiota-derived metabolites, including the two best characterized protein-bound uremic retention solutes, indoxyl-sulfate (IS) and *p*-cresyl sulfate.

Medium cut-off membrane (MCO) dialyzers remove not only medium-sized middle molecules, such as β_2_-microglobulin, but they also clear larger middle molecules associated with adverse outcomes in uremic patients, such as free immunoglobulin light chains [20], more effectively than high-flux HD [21]. However, it can be expected that the clearance of medications will also be higher in the case of MCO dialyzer use. Vancomycin is an antibiotic widely applied in HD patients for the treatment of a variety of infections. In a pharmacokinetic study, Allawati et al. [22] compared the clearance of vancomycin during HD using the MCO membrane Theranova® with that of the standard high-flux membrane Revaclear. They found that vancomycin clearance was higher with MCO-HD compared to high-flux HD, and concluded that the application of vancomycin during the last one to two hours of each MCO-dialysis is necessary to retain therapeutic concentrations.

We hope that this Special Issue will cohesively increase the insights into the complex mechanisms of uremic immune dysfunction for the interested reader.
